# Patient satisfaction with pharmacist-administered COVID-19 vaccines in Poland: a survey study in the vaccination centres context

**DOI:** 10.1186/s12913-022-08720-w

**Published:** 2022-11-11

**Authors:** Piotr Merks, Anna Kowalczuk, Alexandre Wong, Kevin Chung, Urszula Religioni, Dariusz Świetlik, Katarzyna Rotmans-Plagens, Jameason Cameron, Katarina Fehir Sola, Justyna Kazmierczak, Eliza Blicharska, Regis Vaillancourt, Agnieszka Neumann-Podczaska

**Affiliations:** 1 Faculty of Medicine, Collegium Medicum, Cardinal Stefan Wyszyński University, Warszawa, Poland; 2 National Institute of Medicine, Warsaw, Poland; 3 Pharmacy Department, Children’s Hospital of Eastern Ontario, Ottawa, Canada; 4 Collegium of Business Administration, Warsaw School of Economics, Warsaw, Poland; 5 School of Public Health, Centre of Postgraduate Medical Education of Warsaw, Warsaw, Poland; 6 Department of Biostatistics and Neural Networks, Medical University of Gdansk, Gdansk, Poland; 7 Hipolit Cegielski State University of Applied Sciences, Gniezno, Poland; 8 Pharmacy of Bjelovar, Petra Preradovića4, 43000 Bjelovar, Croatia; 9 Zdrowit sp. z o.o., Pharmacy Chain, ul, Diamentowa 3, 41-940 Piekary Śląskie, Poland; 10 Department of Analytical Chemistry, Faculty of Pharmacy with Division of Medical Analytics, Medical University of Lublin, Chodźki 4a St., 20-093 Lublin, Poland; 11 Chair and Department of Palliative Medicine, Poznan University of Medical Sciences, Poznan, Poland

**Keywords:** Vaccination, Vaccination centres, Pharmacist, Patient, COVID-19

## Abstract

**Background:**

Since 2021, pharmacists in Poland have been authorised to administer vaccinations against COVID-19, which is of particular significance in the efforts towards preventing the spread of the pandemic. The primary objective of this study was to evaluate the patients’ satisfaction with delivering vaccinations through national vaccination centres.

**Methods:**

This study was conducted in 2021. The research tool was an anonymous questionnaire distributed to patients after vaccination. The questionnaire was developed specifically for the purpose of the study. Ultimately, 628 patients participated in this study.

**Results:**

Nearly 97% of the respondents agreed that the administration of vaccinations by pharmacists had been convenient, and pharmacists possessed the relevant skills to provide this service. Almost 90% of the respondents expressed their readiness to be vaccinated by pharmacists again. Nearly all the respondents indicated that pharmacists should also provide other vaccinations.

**Conclusions:**

Patients in Poland have a positive attitude toward vaccinations administered by pharmacists in national vaccination centres.

**Supplementary Information:**

The online version contains supplementary material available at 10.1186/s12913-022-08720-w.

## Background

Since 2021, pharmacists in Poland have been authorised to administer vaccinations against COVID-19, which is of particular significance in view of the spreading pandemic. This was the first opportunity for Polish pharmacists to provide a vaccination service. At the end of December 2021, more than 3.5 million people in Poland have been infected with COVID-19 (about 10% of the population), and nearly 80 thousand people have died [1]. The World Health Organisation (WHO) considers vaccinations against COVID-19 the most effective way to combat this disease [2]. To date, about 54% people in Poland have been fully vaccinated (as of the end of November 2021) [1]. For this reason, further efforts to increase the vaccination coverage level in Poland in order to prevent the spread of the pandemic seem essential.

Protective vaccinations administered by pharmacists have been common for many years in many countries worldwide, e.g. Great Britain, Canada and the USA [3–5]. Literature data indicates that the number of vaccinations provided by pharmacists has been on the rise because of easy access of pharmacies, which may have an impact on the overall vaccination coverage level [6]. Vaccinations offered by pharmacists increase the availability of this service [7] as pharmacists constitute one of the largest groups of medical professionals in Poland (0.77 per thousand inhabitants) [8]. Previous studies carried out in Poland show the readiness of pharmacists to administer vaccinations. Pharmacists believe that community pharmacies are easily accessible and pharmacists have the necessary skills to provide vaccination service [9].

In Poland, pharmacists with appropriate qualifications are authorised to qualify for and administer vaccinations. These qualifications are acquired at a two-stage course that includes theoretical and practical training. The theoretical part concerns medical (e.g. indications and contraindications for vaccinations) and technical/informatical (correct digital documentation of vaccinations) issues. In the first weeks after obtaining the right to administer vaccinations, several thousand pharmacists in Poland completed this course [10].

Since the COVID-19 pandemic, vaccination centers have been established in Poland. This was to increase the availability of the vaccination service for patients and to vaccinate as many patients as possible in the shortest possible time. Until now, vaccinations were performed in medical institutions, mainly by nurses.

Given the above, the primary objective of this study was to evaluate patient satisfaction with vaccinations administered by pharmacists in national vaccination centres, while a secondary objective was to indicate the influence of the basic sociodemographic variables on patient satisfaction with the vaccinations.

## Methods

This study of the opinions of patients was carried out in the period of May to September 2021. The research tool was an anonymous questionnaire developed for the purposes of this study consisting of 4 questions on patient satisfaction with vaccinations, 3 questions on the symptoms of COVID-19 and any adverse events following immunisation, as well as personal data. These required a single-choice answer from ready multiple-choices (apart from 2 open questions). The questionnaire developed for this study is provided as Additional file 1.

The questionnaire was distributed to patients after vaccination. Each patient vaccinated by a pharmacist received the questionnaire immediately after vaccination. The inclusion criteria for patients were: age over 13 and literacy. Ultimately, 628 patients took part in the study. Patients filled in the questionnaire in the waiting room and gave it to the indicated person. Each patient who received the questionnaire completed it. If the respondent was a minor (a person under 13 years of age), consent to the study was obtained from the parent who accompanied the child to the vaccination.

The study was approved by the Bioethics Committee of the National Geriatrics, Reumatology and Rehabilitation Institute in Warsaw (decision no. KBT – 3/3/2021).

### Statistical methods

The statistical analyses have been performed using the statistical suite StatSoft. Inc. (2014). STATISTICA (data analysis software system). version 12.0. www.statsoft.com and Excel.

The quantitive variables were characterized by the arithmetic mean of standard deviation or median or max/min (range) and 95% confidence interval. The qualitative variables were presented with the use of count and percentage.

In order to check if a quantitive variable derives from a population of normal distribution the W Shapiro-Wilk test has been used. Whereas to prove the hypotheses on homogeneity of variances Leven (Brown-Forsythe) test has been utilized. In order to determine dependence, strength and direction between variables, correlation analysis was used by determining the Pearson or Spearman’s correlation coefficients. In all the calculations the statistical significance level of *p* = 0.05 has been used.

### Sample

The study group distribution was 52% women and 48% men. The mean age of the respondents was 31.8 years. The largest group of respondents lived in cities above 500 thousand inhabitants (80.5%). Slightly more than half of the respondents declared being single (53.2%). Over 61% of the respondents had higher education (Table 1).

**Table 1:** Characteristics of the study group

	Study group (***n*** = 628)
**Age**	
mean (SD)	31.8 (12.3)
range	13-97
median	30
**Sex**	
Male	295 (48.0%)
Female	319 (52.0%)
**Education**	
primary	56 (9.1%)
basic vocational	7 (1.1%)
secondary	173 (28.0%)
higher	382 (61.8%)
**Marital status**	
married/in a partnership	268 (43.6%)
divorced	20 (3.3%)
single	327 (53.2%)
**Place of residence**	
village	61 (9.8%)
city < 200 thousand inhabitants	50 (8.1%)
city 200-500 thousand inhabitants	10 (1.6%)
city above 500 thousand inhabitants	500 (80.5%)

## Results

### Opinions on satisfaction with vaccinations through vaccination centres

The respondents largely agreed that vaccinations administered by pharmacists were convenient (the sum of responses ‘Agree’ and ‘Strongly agree’ was 96.9%). Nearly 97% of the respondents were of the opinion that pharmacists possessed the appropriate skills to administer vaccinations. Almost 90% of the respondents indicated that they would get vaccinated by pharmacists if it were possible. A similar percentage of respondents would like pharmacists to also be able to administer other vaccinations (Table 2).

**Table 2:** To what extent do you agree with these statements (1 – strongly disagree; 5 – strongly agree)?

Statement	Number of patients (***N*** = 628) ***N*** (%)				
	Strongly disagree (1)	Disagree (2)	Neither agree nor disagree (3)	Agree (4)	Strongly agree (5)
Getting vaccinated by a pharmacist is convenient	7 (1.1%)	2 (0.3%)	10 (1.6%)	29 (4.7%)	568 (92.2%)
In my opinion, the pharmacist possessed the relevant skills to administer vaccinations	7 (1.1%)	1 (0.2%)	12 (2.0%)	31 (5.0%)	564 (91.7%)
If possible, I will get vaccinated by a pharmacist again	27 (4.7%)	12 (2.1%)	33 (5.7%)	78 (13.5%)	429 (74.1%)
I would like pharmacists to administer other vaccinations in the future	14 (2.4%)	7 (1.2%)	27 (4.6%)	83 (14.1%)	459 (77.8%)

Correlations of the above mentioned issues with demographic variables show that as the age of the respondents increased, they were less likely to agree with the statement regarding the convenience of vaccinations administered by pharmacists (the correlation coefficient *R* = -0.10, *p* = 0.0197). A similar dependency was recorded in relations to the increase in education (the correlation coefficient *R* = -0.10, *p* = 0.0100). In addition, as education increased, the scale regarding the opinion of having the skills to provide vaccinations decreased (the correlation coefficient *R* = -0.10, *p* = 0.0171), while the scale regarding the possibility of being vaccinated again by a pharmacist increased (the correlation coefficient *R* = 0.10, *p* = 0.0324) (Table 3).

**Table 3:** Patients’ opinions about vaccinations - correlations with sociodemographic variables

Statement	Correlations with sociodemographic variables				
	Sex	Age	Education level	Marital status	Place of residence
Getting vaccinated by a pharmacist is convenient	*p* > 0.05	*R* = -0.10, *p* = 0.0197	*R* = -0.10, *p* = 0.0100	*p* > 0.05	*p* > 0.05
In my opinion, the pharmacist possessed the relevant skills to administer vaccinations	*p* > 0.05	*p* > 0.05	*R* = -0.10, *p* = 0.0171	*p* > 0.05	*p* > 0.05
If possible, I will get vaccinated by a pharmacist again	*p* = 0.0109	*p* > 0.05	*R* = 0.10, *p* = 0.0324	*p* = 0.0134	*p* > 0.05
I would like pharmacists to administer other vaccinations in the future	*p* > 0.05	*p* > 0.05	*p* > 0.05	*p* > 0.05	*p* > 0.05

As far as sex is concerned, women were significantly more likely to have their next vaccination administered by a pharmacist (*p* = 0.0109). Married respondents and those living in a partnership were significantly more likely to have their next vaccination administered by a pharmacist (*p* = 0.0134).

In the past 6 months, 11.7% of the respondents developed symptoms of COVID-19 (Table 4) – fever (37%), loss of smell and taste (41.1%), flu-like symptoms (16.4%) and a cough (11.0%). Men significantly more often than women developed symptoms that could indicate COVID-19 (*p* = 0.0192), yet SARS-CoV-2 had been detected in 6 out of 73 respondents with COVID-19 symptoms.

**Table 4:** Information on COVID-19 and vaccinations

Question	Number of patients (***N*** = 628) ***N*** (%)		
	Yes	No	Don’t remember
In the past six months, have you developed any symptoms that could indicate COVID-19?	73 (11.7%)	548 (87.5%)	5 (0.8%)
In the past six months, have you taken sick leave due to these symptoms?	33 (5.3%)	593 (94.7%)	0 (0.0%)
Have you ever had any adverse events following the vaccination?	43 (6.9%)	578 (92.3%)	5 (0.8%)

In the past 6 months, slightly more than 5% of the respondents took sick leave due to coronavirus symptoms, with a mean length of 9.1 days (SD = 15.5).

Nearly 7% of the respondents experienced adverse events following immunisation: a sore arm (25.6%), fatigue (18.6%), headache (16.3%) and fever (16.3%). Statistical analysis showed that women significantly more often than men experienced adverse events following immunisation (*p* = 0.0001). Patients who did not report adverse events following immunisation were significantly older than those who reported these events (*p* = 0.0045).

## Discussion

The establishment of vaccination centers and the obtaining of the authorization by pharmacists to perform vaccination were a milestone in Polish health care. These measures were a response to the spreading COVID-19 pandemic. Therefore, our goal was to check whether Polish patients are satisfied with the vaccination services provided by pharmacists at vaccination centers.

Our study indicates that patients in Poland are of the opinion that getting vaccinated by pharmacists is convenient, and that they feel that pharmacists possess the relevant competencies to provide this service. The vast majority of the respondents reported that they would get vaccinated by a pharmacist again if it were possible. Similarly, patients think that pharmacists in Poland should also be authorised to administer other protective vaccinations.

The percentage of respondents satisfied with vaccinations administered by pharmacists (including convenience and qualifications of pharmacists) was about 97%, which is similar to studies carried out in other countries, e.g. Australia [7], Canada [11] and Estonia [12]. The studies also indicate that people satisfied with vaccinations provided by pharmacists in community pharmacies recommend this service to others, which may increase vaccination coverage levels [13].

Considering the medical data of the respondents, pharmacist engagement in providing vaccinations as part of pharmaceutical care is particularly significant. In our study group, nearly 12% of the respondents developed symptoms that could indicate COVID-19, yet the test confirmed this infection in only a few of them. In the case of symptoms, many of those respondents had the opportunity to receive professional pharmaceutical help, e.g. an appropriate selection of medications in relation to symptoms, which could largely shorten the length of sick leave and thus reduce global economic and social costs. Similarly, pharmacists with extensive knowledge of medications can to a large extent help patients deal with the adverse events experienced by nearly 7% of the respondents.

While some studies indicate that it is mainly women who get vaccinated by pharmacists and the results of studies are differentiated by multiple demographic variables [14], our study group was to a large extent homogenous (similar percentage of women and men, single and married respondents, large age differentiation). Several dependencies regarding the aspects of satisfaction with vaccinations in relation to demographic features were observed. For example, the willingness of receiving the next vaccination from a pharmacist was indicated mainly by women, people with higher education and married respondents. Large-scale studies on this type of dependency may serve for targeting health promotion actions performed by pharmacists at specific groups of recipients in order to achieve the largest effectiveness possible [15].

Considering the number and availability of pharmacists in Poland, it seems that this group of medical staff can greatly impact public health. Pharmacies (often open 24 hours a day, without the necessity to make appointments, open even during the pandemic) are frequently the patients’ first contact points with healthcare staff. For this reason, activities performed by pharmacists, particularly within health promotion and prophylaxis, can largely improve patient health results [16, 17].

Previous data on vaccinations in Poland indicate a very low vaccination coverage level. For example, in the case of flu, it is about 6-7% [18], compared to other countries such as Great Britain, Northern Ireland, Portugal and the Netherlands (60-70%) [19]. The vaccination coverage level against COVID-19 is much higher than in the case of flu (54% have received two vaccination doses). One influencing factor can be the significantly increased availability of vaccinations, which impacts the convenience and decision on getting vaccinated [5, 20]. In the majority of pharmacies and vaccination centres in Poland, vaccinations against COVID-19 are administered until evening hours, while vaccinations against flu were possible exclusively in outpatient clinics during the working hours of a doctor qualifying for vaccinations and a nurse administering vaccinations. Studies carried out in other countries also show that pharmacist engagement in administering vaccinations against COVID-19 can have a significant influence on stopping the pandemic in Europe [21].

The willingness of pharmacists in Poland [9] and other countries [22, 23] to expand pharmaceutical services is of great importance in this respect. When implementing or expanding pharmaceutical services with vaccinations administered by pharmacists, one should also be aware of the barriers that can impact the effectiveness of implementation of these services in healthcare systems: barriers associated with the organisation and administration of vaccinations, legal barriers, and barriers connected with settlement of vaccination services [3, 24, 25]. The necessity of practical training in order to acquire vaccination skills is also indicated [26]. Efforts made to reduce these barriers can largely influence the perception of pharmacists as people competent to administer vaccinations, which will increase the availability of these services, relieve the burden on medical staff, as well as provide patients with accurate, precise and reliable information on products. These activities can also increase the number of vaccinations and thus play an important role in public health protection.

### Limitations

The results of our study indicate high patient satisfaction with the services provided by pharmacists in vaccination centers. Nevertheless, it is possible that the results may not represent a material impact if a larger, random sample, was used.

Another limitation is the lack of knowledge of how many people in Poland were vaccinated by pharmacists. Therefore, we do not know which sample of patients we included in our study.

## Conclusions

Patients in Poland are satisfied with vaccinations administered by pharmacists who in their opinion are properly qualified for this service. These results are of great importance, taking into account the fact that Polish pharmacists have recently been able to perform the vaccination service. The vast majority of the respondents indicated their trust in pharmacists and their willingness to have their next vaccination administered by a pharmacist. Pharmacists are a large and accessible professional group ready to provide further vaccinations. Expanding pharmaceutical services with vaccinations administered by pharmacists in Poland can impact the overall vaccination coverage level and relieve the burden on other medical professions.

## Supplementary Information

**Additional file 1.** Questionnaire

## Data Availability

All data are available from the corresponding author.

## Abbreviation

WHO: World Health Organisation
