# Supplementary material for: Patient satisfaction with pharmacist-administered COVID-19 vaccines in Poland: a survey study in the vaccination centres context
Source: BMC Health Serv Res. 2022 Nov 11;22:1339. doi: 10.1186/s12913-022-08720-w (PMC9652033; doi:10.1186/s12913-022-08720-w)
Supplement: Supplementary file 1 — Additional file 1. Questionnaire [file 12913_2022_8720_MOESM1_ESM.doc]

Dear Patient, we kindly invite you to participate in the survey on satisfaction with the vaccination service provided by a pharmacist. The research is anonymous and its results will be used for research purposes only.

**DEMOGRAPHIC DATA**

**1.Gender:** ☐M ☐F **2. Year of the patient's birth**: _________

**3. Education:** **4. Marital status**

☐ primary ☐ bachelor / maiden

☐ vocational ☐ married / married / in a partnership

☐ secondary ☐ divorced

☐ higher ☐ widower / widow

5. **Place of residence**

☐ village

☐ city < 200 thousand residents

☐ city ​​200-500 thousand residents

☐ city ​​>500 thousand residents

**VACCINATION SATISFACTION**

Please indicate to what extent you agree with the following statements by ticking an answer ([
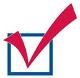
](http://www.google.ca/imgres?imgurl=http://mustquitsmokingnow.com/check_mark.gif&imgrefurl=http://mustquitsmokingnow.com/&h=311&w=320&sz=4&tbnid=GQlxhvtL5u10KM:&tbnh=115&tbnw=118&prev=/images?q=check+mark&zoom=1&q=check+mark&hl=en&usg=__C6VDeBAJCoRfwzCRaCw2mgH174E=&sa=X&ei=PAQtTcqPKpP4sAO_kcHNBg&ved=0CDMQ9QEwBQ)) in the appropriate box, where: 1- I disagree completely, and 5- I strongly agree.

| 1. Receiving a vaccination done by a pharmacist is convenient. |  |  |  |  |  |
| --- | --- | --- | --- | --- | --- |
|  | 1 | 2 | 3 | 4 | 5 |
| 1. In my opinion, the pharmacist had the skills to perform the vaccination. |  |  |  |  |  |
|  | 1 | 2 | 3 | 4 | 5 |
| 1. If it is possible, I will also do the next vaccination at the pharmacist / pharmacy. |  |  |  |  |  |
|  | 1 | 2 | 3 | 4 | 5 |
| 1. I would like pharmacists to perform other vaccinations in the future. |  |  |  |  |  |
|  | 1 | 2 | 3 | 4 | 5 |

**ADDITIONAL INFORMATION**

1. In the last six months, have you experienced symptoms suggesting COVID-19 (high fever, flu-like symptoms, loss of smell / taste, etc.)?

☐ yes – what? …………………………..

☐ no

1. In the last six months, have you been on sick leave due to this type of symptoms?

☐ yes – if yes, how long? …………………(number of days)

☐ no

1. Have you ever had an adverse reaction after vaccination?

☐ yes – what?...........................................

☐ no

Thank you for completing the survey!
